# Multimodal co-therapy for unaccompanied minors: a qualitative study

**DOI:** 10.1186/s13034-022-00518-2

**Published:** 2022-11-07

**Authors:** Sélim Benjamin Guessoum, Sevan Minassian, Pauline de Staël, Fatima Touhami, Madeline DiGiovanni, Rahmeth Radjack, Marie Rose Moro, Laelia Benoit

**Affiliations:** 1 Department of Child and Adolescent Psychiatry, Assistance Publique ‐ Hôpitaux de Paris (APHP), Cochin University Hospital, 97 Boulevard Port-Royal, 75014 Paris, France; 2 Université Paris Cité, PCPP, Paris, France; 3 Université Paris-Saclay, UVSQ, Inserm U1018, CESP, Team DevPsy, Villejuif, France; 4 Child Study Center, Yale School of Medicine, New Haven, USA

**Keywords:** Unaccompanied refugee minors, Migration, Psychotherapy, Narrativity, Trauma, Transcultural psychiatry

## Abstract

**Background:**

Unaccompanied refugee minors—or unaccompanied minors—are children and adolescents who have been separated from parents and other relatives and are not being cared for by an adult. Unaccompanied minors are a vulnerable population, with numerous stressors and complex psychiatric symptoms necessitating specialized mental health care. This study explores patients’ experiences of a Multimodal Co-Therapy for Unaccompanied Minors (MUCTUM), which encompasses cultural, biological, narrative & institutional approaches to care.

**Methods:**

MUCTUM is a co-therapy program for unaccompanied minors, with a psychiatrist, psychologist, native-language interpreter, and caseworker for each patient. In this qualitative study, we interviewed adolescents about their experiences with MUCTUM and analyzed these semi-structured interviews using a phenomenological framework (Interpretative Phenomenological Analysis).

**Results:**

Qualitative analysis of 16 interviews discovered that unaccompanied minors felt misunderstood before participating in MUCTUM, describing a sense of strangeness and loneliness in relation to psychiatric symptoms. Several youths experienced triple stigmatization: of being unaccompanied minors, of suffering from psychotrauma, and of being mental health patients. We further describe three overarching domains that inform on MUCTUM support to unaccompanied minors: (1) A safe space for unaccompanied minors; (2) Helpful interventions during therapy; and (3) Narrating one’s story can “set us free” if guided carefully by care providers.

**Conclusion:**

This study suggests that MUCTUM therapy may efficiently support unaccompanied minors’ mental health by acknowledging their hierarchy of needs. Psychotherapeutic strategies include creating a safe place, providing culturally appropriate care and patient-centered therapy, addressing concrete problems, supporting relationships, and making use of *limited reparenting* in therapy. Delayed and progressive inquiry about traumatic events may be beneficial. Replication of these findings and their field application is warranted.

**Supplementary Information:**

The online version contains supplementary material available at 10.1186/s13034-022-00518-2.

## Introduction

Unaccompanied refugee minors—or unaccompanied minors (UM)—are children and adolescents who have been separated from both parents and other relatives and are not being cared for by an adult [1]. The refugee population included an estimated 153,300 UM worldwide in 2019 [2]. UM are a vulnerable group of refugees and experience a high rate of psychiatric symptoms with intricate post-traumatic, transcultural, family, educational, and legal struggles [3]. The cultural and political backgrounds of UM's native countries are often very different from those of their host countries, and these cultural differences can confuse caseworkers and care providers, who are often untrained in caring for adolescents in a migratory context.

UM frequently exhibit mental health problems, with a high prevalence of anxiety (10–85%), depression (12–76%), post-traumatic stress (17–85%), and behavioral problems, and these symptoms are often chronic [4–8]. UMs’ post-traumatic symptoms are complex, and standard nosographies capture only partial features of their clinical expression [9]. Moreover, some adolescents face specific challenges: debt, prostitution, human trafficking, acute stress related to the asylum process [10], and the risk of “aging out” of the Child Status protection. Such factors aggravate the psychological vulnerability of these adolescents, but UM can have positive long-term outcomes if they are provided with appropriate health and psychosocial protection [2].

Providing appropriate care for UM is a challenge for healthcare institutions due to cross-cultural factors. UM’s personal trajectories can affect the acquisition of certain competencies in their native culture and may create difficulties with acculturation [5]. Cultural misunderstandings between UM and care teams can create barriers and misunderstandings at the educational, behavioral, psychological and emotional levels. Regarding psychiatric care, high rates of misdiagnosis are observed in migrant populations [11]. Therefore, there is a need for specialized mental health care for UM, with cross-cultural competencies and a focus on the migration experience and complex trauma.

To date, few studies have been published on therapies for UM [12–14], requiring further evaluation of informed strategies for these young patients. Quantitative assessments of interventions designed for UM have failed to provide significant findings, due to small samples and heterogeneous populations. Similarly, migrant patients tend to be excluded from prospective cohorts particularly because of language barriers, leading to insufficient scientific knowledge on early mental health care for these minority groups [15, 16]. Qualitative research appears an effective framework to overcome this challenge, as it highlights the singularity of individual views and helps refine patient-centered approaches [17]. We designed this qualitative study to address another shortcoming in regard to UM’s mental health care: most standardized mental health screening tools did not undergo transcultural validation, are biased if used with interpreters, refer to concepts that are hardly understood by some UM, and are not always culturally appropriate [14].

In the Adolescent Medicine department of Cochin Hospital, Paris, France [18], a specific co-therapy was created to meet the special needs of UM, titled Multimodal Co-Therapy for Unaccompanied Minors (MUCTUM). It consists of monthly consultation with a psychiatrist, a psychologist, an interpreter-mediator, a social work caseworker, and the patient. Every meeting provides a combination of approaches: the psychiatric and psychological assessment, the transcultural psychiatry approach, the pharmaco-biological approach through medications, the institutional approach with the participation of the UM’s caseworker, and a narrative approach. The objective of this study is to explore patients’ experiences of MUCTUM to identify the possible beneficial features of this set-up and possible shortcomings, share this knowledge, and improve interventions for UM.

## Methods

### Multimodal co-therapy description

#### Set-up and first consultations

MUCTUM operates monthly, or weekly during acute phases. The team includes ethnically diverse providers and consists of a psychiatrist, a psychologist, the caseworker assigned to the adolescent, and an interpreter. The interpreter is a professional translator who is also trained in cultural mediation and attends every session [19]. MUCTUM aims to provide a pluralist approach in which mental health providers efficiently integrate the psychiatric and psychological assessments and interventions including biological, institutional, transcultural and narrative approaches [3, 20, 21].

**Fig. 1:**
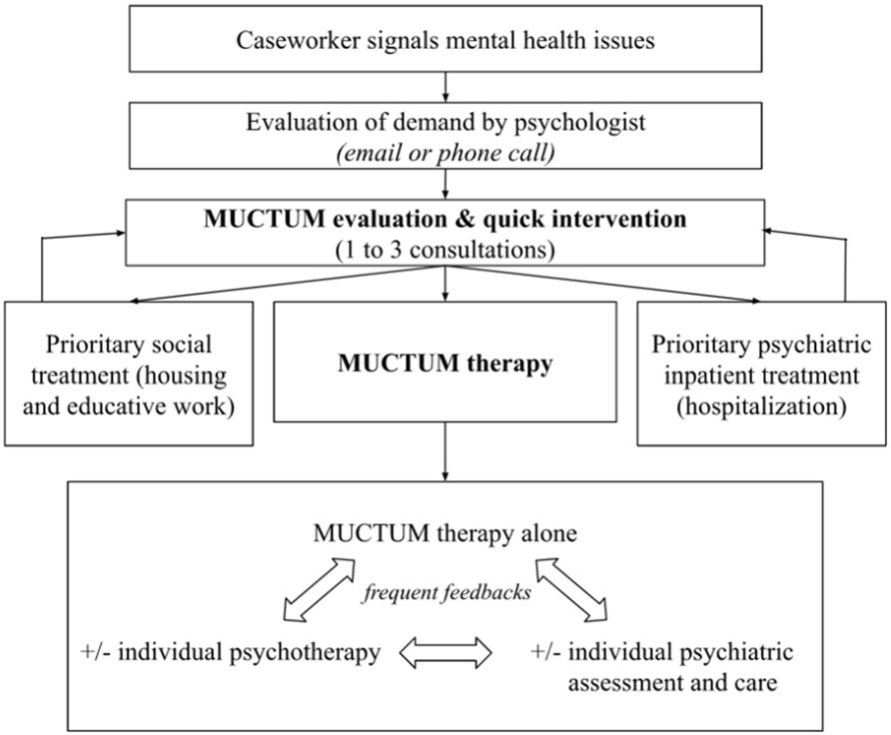
MUCTUM patient-centered treatment approach procedure

During the first session, care providers introduce themselves and the purpose of MUCTUM. Care providers explicitly welcome the patient (“we are happy to receive you, be welcome”), and explain the set-up and the “rules” of the program (confidentiality, kindness, consistent interpreter presence, and ability to speak in both languages whenever). Up to three sessions are dedicated to creating a safe place for the patient, psychiatric and psychological assessment, and first-line therapeutic actions. The remainder of MUCTUM is implemented following the course described in Fig. 1.

First-line therapeutic actions include addressing physical symptoms (including pain and sleep problems) and active communication with other professionals caring for the UM (social workers, caseworkers, nurses, etc.). These first-line actions help build a therapeutic alliance by showing the UM that care providers can solve concrete problems, give priority to what the youth sees as a priority, and restore a feeling of security in his/her daily life environment.

#### Biological approach

Care providers accomplish the usual biological approach by performing a standard psychiatric assessment, but the multimodal emphasis allows psychiatrists to include social factors. Facilitated by the caseworker and the interpreter, care providers are therefore able to relate the UM’s symptoms to social factors and respond accordingly, rather than having a solely pharmacological approach.

#### Institutional approach

An institutional approach is accomplished by the caseworker, who provides a link with the Children’s Foster Home, and who can help address the patient’s behavior, daily life and school problems, and administrative aspects.

#### Transcultural approach

In addition to speaking the UM’s native language and being from the patient’s cultural group, the interpreter is preferably trained as a cultural broker [19]. This allows for a better understanding of the patient’s needs and suffering by clarifying culturally-coded representations and symptoms, so that providers can give appropriate care. The interpreter is a link with the native country, ancestors, and childhood, and allows the team to navigate linguistic nuances and cultural misunderstandings.

#### Narrative approach

The co-therapy model includes a narrative approach: Narration is based on positive life events, positive attachment figures, good memories in the native country (i.e., feasts, food), personal affiliations (i.e. namesakes, parents, grandparents), and verbalization of the UM’s competencies. For example, some adolescents initially deny ever attending school, but are then able to clarify that they acquired some education at religious school. UMs narrate their migratory journey, including adverse life events if comfortable doing so, and set the pace of this sharing process. Being allowed to narrate only positive life events for several sessions promotes a feeling of safety in the therapy setting.

Narration is supported by two exercises that materialize the patient’s journey. First, in the “circles of time,” the patient draws three circles, one for the past, present, and future, and attributes colors to each. The UM then explains why they drew, shaped, and colored the circles in such a way, as a support of narration of their path. Second, in the “three objects,” the patient is invited to bring (or think about) three objects that symbolize past, present, and future, to support the narrative process [21].

### Qualitative research description

This qualitative monocentric study follows the consolidated criteria for reporting qualitative research (COREQ, Additional file 1 Materials) and has been approved by the Ethics Committee of Paris University (CEEI/IRB n° 00,012,021–57). We provided clear oral and written information about the study and obtained verbal and written consent.

#### Population, data collection and analysis

We recruited volunteer participants through purposive sampling among UM who attended MUCTUM therapy in Cochin University Hospital, Paris, France. We stopped recruiting new patients when data saturation was reached, meaning that the analysis of new interviews no longer provided new findings. Three trained researchers conducted semi-structured interviews, either face-to-face or online. Researchers introduced themselves to participants before the day of the interview, and informed UM of the research purpose. Interpreters participated in the interview as needed. The interview guide was adapted iteratively during the study and included sensitizing questions about the following topics: the representation of psychiatric symptoms and care in their home countries and the host countries, what happens and what patients experience during therapy, how patients talk about themselves, the role of the caseworker and interpreter, and how family issues are discussed during therapy. The interviews were audio recorded, anonymized, and transcribed, then analyzed using interpretative phenomenological analysis [22], a method used to understand how people subjectively perceive and make sense of an experience [23]. In this method, researchers read the interviews several times and then annotate and code them in order to identify emerging themes. To ensure a rigorous “triangulation” process [22], three researchers (PDS, SBG, SM) analyzed each interview independently and searched for connections between these emerging themes. Reflexivity was discussed in team meetings, considering that some researchers were part of the hospital institution, and others originated from other teams and countries. Then, we compared the results between interviews to identify recurring themes and integrate new emerging elements. The team (PDS, SBG, SM, LB) then constructed a comprehensive description of participants’ experiences. Themes were organized into overarching domains, and supported by specific quotes from the interviews.

## Results

One hundred and eighteen patients participated in MUCTUM from June 2016 to April 2022. Participants were aged 11 to 17 yo and came from the following areas: Western Africa (Mali, Ivory Coast, Guinea, Senegal), Central Africa (Nigeria, Congo, Congo Kinshasa, Somalia), Northern Africa (Morocco, Algeria, Tunisia), Asia (Afghanistan, Pakistan, Bangladesh), and Europe (Albania). Most participants suffered from anxiety, depressive, or post-traumatic disorders, with a few experiencing psychotic disorders and addictive disorders. Sixteen interviews were conducted from April 2021 to April 2022, ranging from 20 min to one hour. Participants’ characteristics are described in Table 1. Analysis revealed that participants described strangeness and loneliness in relation to psychiatric symptoms, often feeling misunderstood before participating in MUCTUM. Several youth experienced triple stigmatization: of being UM, of suffering from psychotrauma, and of being mental health patients. Some reported feeling suspicious of care providers, due to negative cultural representations of mental health care or limited background knowledge of mental illness. As therapeutic alliance built up, participants felt increasingly helped by the consultations. They no longer experienced suspicion towards care providers, but rather trust. We identified three themes that reflect how UM feel supported, hoping to further improve caregivers’ interventions for UM: (1) A safe space for unaccompanied minors; (2) Helpful interventions during therapy; and (3) Narrating one’s story can “set us free'' if guided carefully by care providers. Themes and subthemes are summarized in Table 2.

**Table 1:** Sociodemographic characteristics of participants

	Age at interview	Gender	Disorder	Birth country	Mother tongue	Duration of care (months)
n°1	17	♂	PTSD	Guinea	Susu	> 12
n°2	18	♀	Eating disorder unspecified, depression, PTSD, post-traumatic amnesia	Somalia	Somali	> 12
n°3	18	♂	PTSD	Ivory Coast	Dyula	> 12
n°4	17	♂	PTSD, suicidality	Burkina Faso	Mooré	> 12
n°5	22	♀	PTSD, depression	Ivory Coast	French	> 12
n°6	17	♂	Depression, suicidality	Mali	Soninke	< 6
n°7	20	♂	Depression, social withdrawal	Guinea	Fula	> 12
n°8	17	♂	PTSD, depression	Cameroun	English, French, (Banyang, Pidgin, Sawa)	6
n°9	18	♂	Depression & anxiety disorder	Mali	Soninke, (Bambara)	> 12
n°10	19	♀	Externalizing disorder, PTSD	Nigeria	Urhobo, Pidgin, English, (Edo)	> 12
n°11	17	♂	Psychotic depression, psychosomatic symptoms, (drepanocytosis)	Guinea	Jakhanke	< 12
n°12	16	♀	PTSD	Congo Kinshasa	Lingala	6 to 12
n°13	17	♂	Anxiety disorder, dissociative disorder	Congo Kinshasa	Lingala	< 6
n°14	16	♀	PTSD, depression	Ivory Coast	Koroka, (Bambara, Dyula)	< 6
n°15	18	♀	Depression, PTSD	Nigeria	Pidgin	> 12
n°16	17	♂	Schizophrenic disorder	Guinea	Fula	< 6

Interpreters took part in interviews for participants n° 1, 3, 11, 16

*PTSD* Post Traumatic Stress Disorder

**Table 2:** Themes and subthemes

A safe space for unaccompanied minors	a. A secure therapeutic framework b. The interpreter prevents misunderstandings and is a reassuring link with the native country c. A support to caseworker-youth relationship
Helpful interventions during MUCTUM therapy	a. Behavioral guidance promotes feelings of safety, self-confidence, and self-care practices b. Care providers facilitate links with the other institutions c. The work on family issues d. Cultural focus enables traditional care implementation e. Help with medications
Narrating one’s story can “set us free” if carefully guided by care providers	a. Unaccompanied minors valued a delayed, progressive, and fractionated narration b. Care providers provide active, participative and empathetic listening to narration c. Sharing one’s story is “painful” but “sets us free”

### A safe space for unaccompanied minors

#### A secure therapeutic framework

The perception that caregivers were compassionate, engaged in care, and steadily present despite repeated missed sessions was cited by participants as a major factor in building therapeutic alliance. Participants considered their care providers “trustful,” “skilled,” “deeply caring,” “generous,” knowledgeable on their culture, and always providing “thorough explanations.”

*" I can have problems in my life and I call Mrs. X on her number. It's really... I don't know about the others, but for me, it's not only a follow-up at the hospital, it's really a follow-up that was really very thorough (...) I improved a lot of things, like anger. I, I worked with that with Dr. Y... anger and everything.... It was something that I couldn't handle... It allowed me to... really deal with some of the things about myself that I didn't like…”*

According to UM, care providers offered much-needed guidance, giving advice and helping to find concrete solutions to daily life problems. They were perceived as role models, and even compared to a parent by some participants, “like a mother.” Several participants reported having the personal phone numbers of care providers (which is unusual in France) in case of crisis: “I could call or send a message (…) and that's what helped me even more, that made things easier for me.” All participants stressed that feeling welcomed at the first session had been essential: “The first time I came, it was great, I felt at ease just like at home.” Laughter was a notable event during the first meeting: "The first day, I was with my caseworker. It worked out well. [Care providers] had a good laugh with me too." According to participants, consistency of care increased adherence, even despite some missed appointments. Said one participant, “Only God knows how many times I didn’t come,” with continuation of MUCTUM contrasting sharply with previous care that canceled follow-ups after only two missed sessions. Care providers became secure references during transfer of care, sharing “knowledge” about participants with new care providers who “don’t know how to handle [them] yet.” Nevertheless, some participants mentioned that the presence of four adults – two care providers, one caseworker and one interpreter – led them to feel “afraid” or “lost” at the beginning. Despite being overwhelming, the presence of several adults was overall seen as helpful to discuss a variety of opinions.

*“At first, I thought it was too much. I said to myself: there are too many people. But then I realized that when there are several people, we talk more. When there are several people, we discuss more, everyone gives their opinion. That's good. That's really good. I think that... Just at the beginning… I thought it was too much, but then I saw that it's better, when there are several people, each one speaks. They also, they talked a little bit about themselves. That's good.”*

#### The interpreter prevents misunderstandings and is a reassuring link with the native country

Participants perceived interpreters as instrumental in achieving full understanding of their identity, origins, language, and culture. Interpreters prevented possible misunderstandings (“so that I don't understand the opposite meaning”) and helped overcome language barriers.

*“With them, I was not alone, we were several. In addition, there was someone who spoke..., who interpreted, who was there, who knows me well, who knows where I come from. He knows the place, he knows my culture and everything.”*

Participants also valued interpreters’ ability to help them articulate difficult subjects to the care providers.

*"I can explain to him what I have. I speak French but I don't really speak, there are things I don't know in French. With him, I can explain and he can understand me. I don't know how to tell you. (...) Often, when I spoke with him in my language, when I explain it to him, the explanation he gives... If it had been me, it would not be the same explanation. He knows the French language well. He explains well, in fact. He explains well compared to me and I wouldn't have explained well like him."*

For many, interpreters illustrated a bond with the native country, especially for UM who had not seen “anyone from the same country” since arriving in the host country.

*“I am of Somalian origin and I am in France since I was little and also when I arrived I did not see Somalian people. So when I see the interpreter every time here it was a pleasure to see a person from the same country as me (...) I felt sometimes that I spoke the same language and all that sometimes I asked questions about my country because I was little when I left my country (...) [the interpreter spoke] about the cultures of the villages, all that, all that, all the things, the food to eat”*

#### A support to caseworker-youth relationship

Some participants identified caseworkers as a reassuring support during sessions.

*"In fact, at the consultation when I go there, when they send me to the consultation, but sometimes when I arrive at the consultation, when we speak, I don't really like it, so I let my caseworker talk, as I really trust her, when we speak. After that, that's when I can talk.”*

However, for some participants, the caseworker’s continuous presence was perceived as intrusive and created a disincentive to talk, especially about very “personal” migratory life events. Some participants criticized the framework as too rigid, preferring to be “asked” if they consented to the caseworker’s presence during therapy.

### Helpful interventions during MUCTUM therapy

Participants identified several helpful interventions during their follow-up.

#### Behavioral guidance promotes feelings of safety, self-confidence, and self-care practices

Participants valued education on coping skills and appropriate behaviors, especially during conflicts.

*" I am someone, I get very, very angry. Even sometimes when I speak, I get angry very, very quickly. So Mrs. Y taught me and I'm learning to breathe and not to get angry very quickly. If someone comes to get me, I will try to speak calmly to these people, so as not to be very aggressive. (...) Once she finds that the situation is very hard, she tells me that I take a breath, I drink a glass of water, that it calms me down and then I speak again. Sometimes when I'm upset or sad, even when I'm at home, I take a drink of water and my throat feels better.”*

Skills that participants cited included emotional regulation, relaxation methods (e.g. breathing exercises, drinking a glass of water), planning (e.g. writing to-do lists in a notebook), and reflection (e.g. “practicing speech in front of [a] mirror”). Participants who had experienced violence and detention also explained how the care providers’ explanations about security in France increased their feeling of safety:

*“It didn't really go away but I evolved a lot on it because before, it was due to everything I had been through, I had the feeling that I was going to be found, no matter what. So that's a little bit of what fueled that feeling too... But then I was made to understand that this is France and all... I was reassured a lot by Mrs X and Dr. Y too and uh I started to let go a little bit, they made me understand that I wasn't going to be attacked overnight in the street... Or kidnapped and everything... So that kind of... calmed me down a bit…”*

One participant explained that she continues to apply these negative affect regulation techniques now that she is a mother, which increases her self-confidence. “I worked [on] self-confidence a lot (…) and it is going very well”.

#### Care providers facilitate links with the other institutions

Care providers facilitate links with the other institutions caring for UM, particularly social workers, who facilitate support for the youth at Children’s Foster Home and school. Although some participants initially expressed displeasure at the presence of caseworkers in therapeutic sessions, most participants said that inclusion of their caseworker in MUCTUM unlocked communication and improved the relationship with the caseworker.

*"I was the one who was really struggling and uh it was really really um... The follow-up that allowed us to tighten up.... Strengthening the ties, no but... Allowed me to have… uh… a discussion with [my caseworker] because having a discussion with her was not possible...*

*Researcher: Yes.*

*Participant: We couldn't understand each other at all.*

*Researcher: Yes.*

*Participant: Because here I was really saying everything I wanted to say and who I really was… [care providers] had the ability to transmit that to another person, so that the person could understand me too…*

*Researcher: Yes.*

*Participant : So uh, [my caseworker] had to go through them to understand how I functioned and me too how she functioned and sometimes we had big meetings where there were people and uh... It was partly thanks to them that I was able to establish a link with my caseworker...”*

For others, MUCTUM provided a place to air discontents about their caseworkers, such as daily conflicts or misunderstandings. Some UM “felt bad” at the Children’s Foster Home, and shared that MUCTUM allowed them to express this opinion. Participants identified that the care providers could help the caseworkers learn how to navigate certain situations more effectively, such as one participant experiencing social withdrawal or mutism:

*“Basically, you know as I said, basically, I didn't talk to anybody. So everybody was like, how do I talk to this individual? (...) So it was complicated for them... And in the end uh... (...) So, since there are certain things that I don't want to tell them about. So I invited them to come and participate in my sessions. That way, they will hear certain things. So, when we go back [home], [if] there are certain things that they don't (...) understand or that kind of thing, they call me, we talk about it, they ask me questions. And I answer, if I feel it... I answer. And so with that, it allows them to know how (...) to talk to me, how, how to handle the situation with me.”*

In another example, one participant’s need for privacy due to his traumatic history was acknowledged, and he obtained access to an individual room. In addition to collaborating with the social workers, care providers use a problem solving approach to address social, educational and legal problems. One participant reported how care providers supported her to pursue her academic studies and advocated for her with social services. Another reported that he felt supported for minority recognition administrative procedures with his lawyer, to whom he “did not say everything” previously because of fear and uncertainty.

#### The work on family issues

Family issues were often discussed during therapy, such as the violent loss of parents, or the family relational difficulties that had led the UM to exile.

*"There were problems with the family. My mother, who was not present, left us long ago. I was with my aunt and her children. Often it was not going well. That's why I left there. There is all that, we talked about all that too, with them."*

In particular, participants valued phone calls and advice from the care team to family back home. In the case of one participant who had previously been sending money home due to being a victim of prostitution, care providers called the participant’s mother to explain that her daughter, now in school with no income, could no longer send money back home:

*"I told all my stories to Mrs. X. In any case, she found some words. She called my mother to explain something to her. She made it clear that I don't work here, that I'm studying, that I won't have enough money, as much as she asks, that I won't be able to finance everything. So my mother, I don't know, but she has trouble understanding, my mother. She had a hard time."*

This communication induced a change in parental expectations, which “relieves more and more” for adolescents seeking reassurance that it is okay to refuse to send money to over-demanding family members. For another participant, interpreters facilitated a conversation with the participant’s mother back in Somalia in order to restore family links, relationships, and childhood memories, which had been erased because of traumatic amnesia: “All my culture (…), when I was a child, lullabies, all this.” For this participant, finding her mother was a “huge relief” and allowed this adolescent to “reconnect to reality.” Now, the ability to call her mother frequently has “changed” and “energized” the participant: “It gave [me a] smile, me who was always sad.”

#### Cultural focus enables traditional care implementation

Cultural etiology and treatment were acknowledged and employed as needed, as cultural etiologies had variable importance for participants across the sample. Most participants did have representations of their conditions in their native culture (e.g. *kénu* in Soninke language, a certain conception of worry, or *undusu*, a “heart discomfort” in Malenke language), or explanations for certain phenomena (e.g. *djodjo*, witchcraft in Nigeria) and their cures. Some had no cultural hypothesis because they had left their country too young and their only solution was to “talk to my parents, and they will take care of me.” Other participants knew traditional etiological explanations, such as possession by spirits, like djinns, but associated djinns with problems more severe than their own. Participants also described various traditional treatments, specific to their home countries, including herbal treatments, massages, reading the Bible and listening to music, reading, and alms ("I buy stuff and give to the poor, give to people, and I ask God to protect me"). Some participants reported that their families in the native country recommended these traditional treatments, which they completed in France or their native country with the support of their MUCTUM care providers, such as prayers, alms, and sacrifices.

*"I had a dream about an old man (…) I had to get out the alms and I managed to call my mother to do that alms and since then it's been fine."*

#### Help with medications

Participants mentioned the value of medications, which reportedly helped with anxiety and sleep problems, but did not elaborate further on this topic.

*“Before… I was awake… every hour… I tried to sleep at night but I couldn’t sleep… I had nightmares even if the door is open. I got out at night like people who walk at night, I sleep but I got out at the beginning. As I spoke to the psychologist and the psychiatrist, it stopped, I took medicine to sleep.*

*Researcher: Okay*

*Participant: And that stopped and even if I have a bad memory, a nightmare, well I still sleep but before I didn't sleep and all that.”*

### Narrating one’s story can “set us free” if carefully guided by care providers

#### UM valued a delayed, progressive, and fractionated narration

Several participants said that the narration of their story at a slow pace was important, once a relationship of trust had been established with care providers. The narration was described as progressive, fractionated, prudent and non-forced:

*"It was a little bit each session. Just a bit, a bit… and then the whole thing and that's it. It wasn't like 'you have to tell us what you've been through right away.'”*

Participants appreciated the care providers' attention to preventing participants from sharing “too much” at once:

*“If we manage to empty ourselves of this, in fact we empty our heads. When I was able to clear my past with Mrs. X, she found solutions, words and things that could calm me down. (...) They know their work well and they know very well that when it goes too far, they also know how to stop the person when it goes too far. So I like that.”*

Consequently, narrating one’s history was not perceived as obligatory, and in fact some participants recalled relief at being explicitly asked not to talk about their story during early stages of therapy, because it was too painful.

*“Participant: As I said earlier, I'm not someone who likes to re-explain that part.*

*Researcher: Yes*

*Participant: But afterwards, it was really a moment when you had to talk.*

*Researcher: Yes.*

*Participant: It was really. They gave me time, they let me... I think it took a year and a half before... That we really got into it. We only talked about myself, my fears, my mother. And then we really went through the process where ... well I had to explain my story to them and that was it…”*

Narration encompassed both positive and negative life events: "I talked about my memories, times that went well and times that didn't go well, too." Some UM, on the contrary, did not narrate adverse life events: "I didn't tell my story to them." Narration was at times difficult for participants whose memories remained mostly “images” rather than easily-verbalized stories.

#### Care providers provide active, participative and empathetic listening to narration

Although this would seem self-evident for most practitioners, participants frequently and independently volunteered these reflections on providers' attitudes during narration of traumatic events, making it a notable theme in UM's experiences. Care providers were described as empathetic and non-judgmental, which contributed to trust and openness. Participants described how narration involved rephrasing by care providers, creating active, participative, and empathetic listening by care providers: "It was really like, ‘I experience the story with you. It helps you to let it go." Care providers with knowledge of the “sea crossing” helped participants feel understood, as did using “images” when they talked.

*"Participant: It was very, very, very hard. Uh to say again what I… I had experienced, uh… You had to respect… and especially that they, when you explained, they illustrated for you.*

*Researcher: That is to say?*

*Participant: In itself... they take examples from you or do as if I tell them, for example, I crossed the sea: they tell you: by boat, the sea? Basically, to make you really live the thing, but in an intense way so that you can free yourself once and for all, and I think it was not bad for me anyway…*

*Researcher: Yes…*

*Participant: Because when I said uh when I took the example of Italy with the prostitution, really it was illustrated to me... And that was really what I wanted to say. So I was led to say what I had inside me without forcing myself.For example, a person can't understand your past, he's not in your shoes, but in fact, he tries to put himself in your shoes and all that, it feels good."*

Care providers helped UM stop feeling guilty and provided explanations that helped participants “talk more openly.” Said one participant, "When I would say that when I was in Italy, I was prostituting, they would say, ‘yes, we know you were forced. How did this happen to you?’”.

#### Sharing one’s story is “painful” but “sets us free.”

 Several participants mentioned a feeling of "liberation" in relation to the therapy, particularly through the ability to "talk."

*"That's the hardest part. But once you get it all out, you feel good about your body. Plus, we open up to people and they give us ideas, advice on how to be able to deal with our past."*

Sharing one’s story was experienced as “painful” ("it makes me sad,” brings back "weird memories") but provided relief and allowed care providers to find solutions. "It feels good to talk about the things that are in your heart. When you've told someone, it's like everything has gone from your heart." Narrating one’s story, though painful, reduced participants’ feeling of pain and loneliness.

*"It hurt and at the same time, when I was talking, that's when I was able to work on it. When I was able to talk to people where at the same time it relieves my heart and it relieves me in my head."*

It was often the first time they shared what they had endured with someone. “That way, at least someone knows.”

## Discussion

In this qualitative study of semi-structured interviews of UM participating in MUCTUM (Multimodal Co-Therapy for Unaccompanied Minors), we explored participants’ experiences of this therapeutic approach: how UM experience mental health problems, what contributes to creating a safe place, what interventions are helpful during therapy, and UMs’ experience of the narrative approach supported by care providers.

**Fig. 2:**
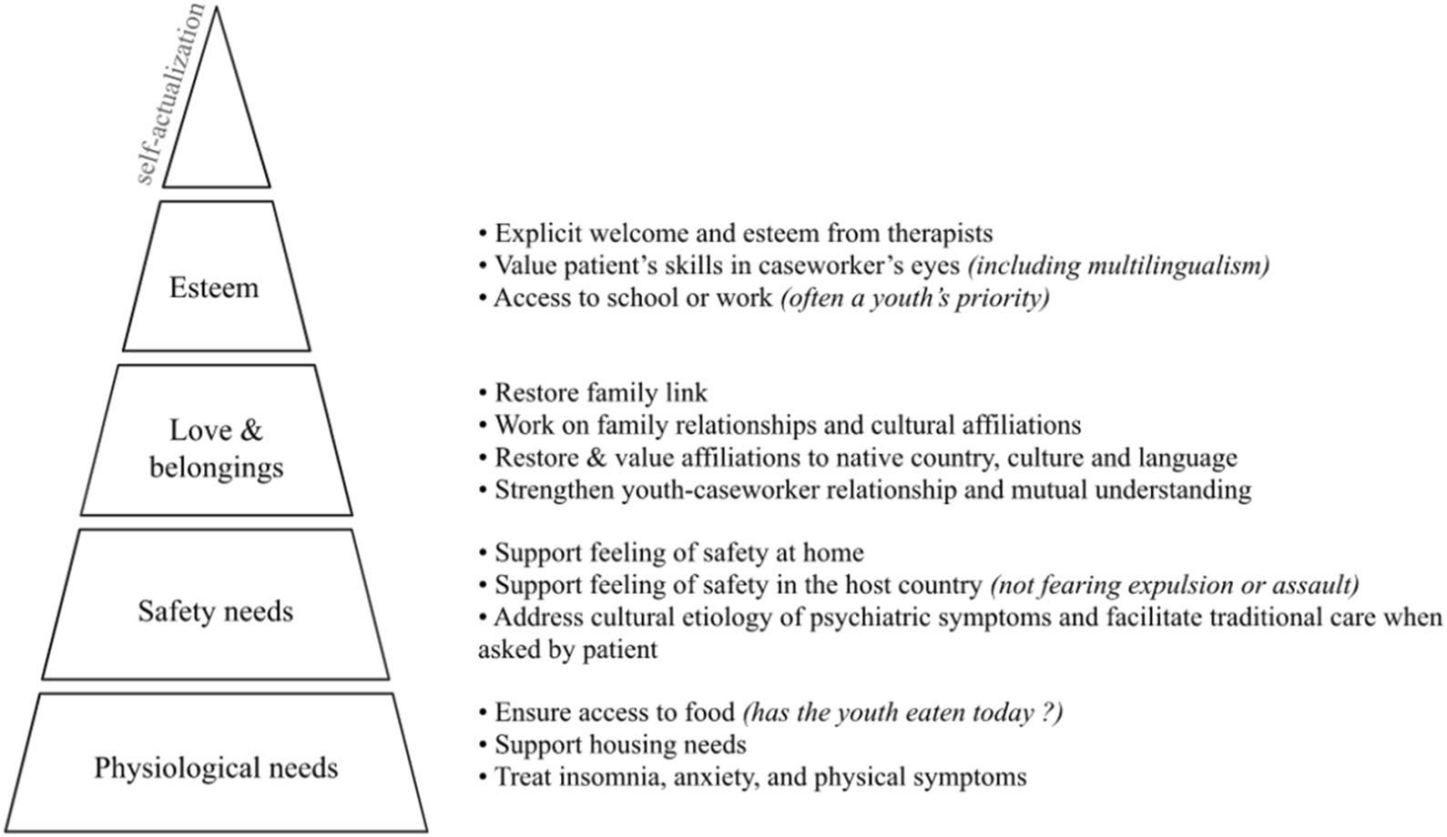
Addressing unaccompanied minors’ needs hierarchically

An important finding is the importance of implementing interventions successively within this multimodal therapy, i.e. the narration of trauma occurs after other interventions. In this sense, MUCTUM follows the fundamental teaching of Maslow’s hierarchy of basic needs [24]. Maslow’s seminal theory on the hierarchy of human needs, motivations and goals reminds care providers to address their young patients’ basic needs before focusing on elaborate therapy goals. As an example, a UM who arrives at therapy after fasting all day will be unable to benefit from cutting-edge trauma-focused therapy if care providers do not feed them first. We present an illustration of the hierarchy of actions for UM in Fig. 2. This is not a rigid hierarchy, as some higher-level needs, such as self-esteem and respect from others, can be supported quickly, restoring a sense of security for UM who have lost confidence in themselves or others. If these principles may appear obvious, they are not always followed in Western health care systems where professionals are encouraged to give their patients only limited time slots and thus rely increasingly on highly standardized interventions. Underrepresented minority groups such as UMs challenge the current expectation of a straightforward and time-limited trauma-focused therapy. In the remaining discussion, we explore how MUCTUM addresses the hierarchy of needs of UMs: creating a safe space, providing patient-centered therapy, solving concrete problems, improving communication with caseworkers, making use of *limited reparenting* in therapy, providing culturally appropriate care and focusing on narrativity of trauma when basic needs have been addressed. Working on family relationships and with families remains relevant, despite the apparent isolation of UMs.

Fear of stigma, suspicion, misunderstandings and negative cultural representations of mental health problems cause denial of mental suffering and are a barrier to mental health care of adolescent refugees. These results are consistent with a qualitative Australian study among 85 refugees aged 13 to 17, in which stigma and misbeliefs about psychiatric conditions were identified as barriers to mental health care [25]. Participants in Anstiss and Ziaian’s study dissimulated disorders to preserve their social status and marriage opportunities. Suspicion towards mental health care has also been found in a UK study among 15 UM, mostly boys from Afghanistan [26]. The study’s title, “This doctor, I not trust him, I’m not safe,” shows how trust in care providers is a prerequisite to experience a sense of safety, which is consistent with the results we described in MUCTUM therapy. The therapeutic alliance then allows UM adolescents to welcome explanatory hypotheses, both from the care providers in their host country and from their families back in their country of origin. This process allows them to make sense of their suffering and to accept treatment.

A warm and reliable setting, including care providers’ long-standing commitment despite missed appointments, is a key ingredient for safety and trust, especially for UM who feel lonely and misunderstood. Other studies describe how to create spaces that allow UM to feel comfortable and valued, to speak openly, and to recover a sense of self [27, 28]. Kohli explores the concept of *safety* for asylum-seeking children: predictable patterns in daily rhythm, going to school, receiving medical care and “finding trust-worthy, reliable and companionable people, adults and peers” [29]. Similarly, creating a safe place for UM is central in MUCTUM.

Patient-centered therapy is a relevant approach for UM, as suggested by our study, because UM have very heterogeneous profiles, and treatment should therefore be based on their unique experience and social situation. This is consistent with Van Es et al.’s study, which advocates for flexibility in treatment approaches for UM [14]. Similarly, MUCTUM provides UMs with a unique program to address and prioritize various problems according to a hierarchy of needs.

Solving concrete problems, e.g. housing, school or medical needs, is essential because it shows UM that care providers understand their priorities. Our participants perceived this problem-solving approach as a sign of their care providers’ competence, leading to increased trust and therapeutic alliance. Other authors highlighted the importance of day-to-day stressors such as missing one’s family, difficulties accessing education, homelessness, sleeping or somatic problems [12, 14]. Together, these findings suggest that addressing physical or social concerns for migrants can be an important first step to their mental health care, in part by initiating a bond with providers [30, 31].

Supporting communication with social workers and foster care providers appeared helpful for this study’s participants. Alto et al. state that nurturing UMs’ foster family environments is central, as enduring conflicts can exacerbate adolescents’ symptoms [32]. The type of placement may also influence UM’s prognosis [33]. Living in foster care is sometimes a protective factor, possibly due to higher level of social support, safety and stability within this setting [34]. Even if complex trauma explains some of the inconsistencies of refugees narratives [35], difficulties in accessing UM’s narrative can raise suspicion of lies and hinder care providers’ and caseworkers’ relationships with UM [36]. Additionally, some adolescents cannot share their personal story for various reasons including fear of insecurity, reprisal, sorcery, or severe trauma. MUCTUM therapy invites caseworkers to learn from UMs’ perspectives by providing them with psychological and cultural skills relevant to the UM. In return, caseworkers can inform care providers on social and educational aspects that improve the team’s overall comprehension of UM’s experience and mental health.

Limited reparenting. Participants reported that they benefited from the reliability of care providers who appeared as secure figures, providing emotional and behavioral guidance. This finding suggests that *limited reparenting* may be a useful component of psychotherapy for UM. Arguably, UMs do not have their core emotional and educational needs met, as they do not have caregivers in the host country. Schema psychotherapy argues that emotional deprivation has three components: deprivation of nurturance (absence of attention, affection, warmth, companionship), deprivation of empathy (absence of listening, understanding, mutual sharing of feelings from others), deprivation of protection (absence of guidance and direction from others) [37]. In the case of UMs, *limited reparenting* by a care provider might partially compensate for this deprivation, provided that appropriate professional boundaries are maintained. According to our participants’ experience, creating “safety, stability, and acceptance” may require care providers to consider sharing their personal phone numbers with patients in case of crisis [38].

Culturally appropriate care is central in MUCTUM, with interpreters who double as cultural mediators. This is consistent with other recent studies, which emphasize the importance of native language and cultural explanation across native and host countries’ cultures, for the young patients and the professionals who care for them [14, 32]. Transcultural psychotherapy is an essential component of MUCTUM, highlighting UMs’ skills acquired in native countries, cultural affiliations to one’s host country, culturally meaningful symptoms, and traditional care if valued by the patient [39]. Transcultural psychotherapy for children and adolescents is currently being assessed with randomized controlled trials [40]. Our results show that UM can give great importance to talking therapy, as proposed in MUCTUM. Although some authors suggest that psychiatric intervention may be deferred until language level of migrant patients has improved [26, 41], we believe that talk therapy can be implemented as soon as an interpreter allows the patient to talk in his/her native language, and as soon as basic needs are provided.

Sharing trauma experiences through narrative can have beneficial effects on health and well-being [42]. In our exploration of how and when trauma should be addressed for UMs, our participants explain that narration of trauma should occur only after several steps of psychotherapy (building trust, concrete problem-solving). These findings suggest that a primarily trauma-focused therapy, as implemented by other programs [14], may be suboptimal for UMs. We argue that when UMs first access care, they are too fragile and trauma symptoms too acute to enable safe and beneficial sharing. Participants in this study felt relieved by focusing on their present (current stressors) and their future (envisioning goals, understanding social, school and administrative procedures of the host countries), rather than traumatic past. Interestingly, this relief in focusing on the present is also experienced by international adoptees, who sometimes prefer to focus on their present life rather than thinking about their country of origin or their biological family [43]. When talking about their past for the first time, participants in our study recalled highlighting positive memories (e.g. important family figures, cultural affiliations to host country, skills acquired in the host country). We argue that addressing present stressors and needs, future expectations, and practical problems are a prerequisite to trauma therapy. Similarly, in their study assessing a primarily trauma-focused therapy for UMs, van Es et al. concluded that some therapies deviated from protocol to address current stressors (e.g. worries about family, school and housing) and current problems (asylum status, recent bereavement), fulfill psychoeducational needs, build trust, lay down a lifeline, and provide translation [14]. Our participants particularly appreciated caregivers’ involvement, participative narration and rephrasing. Such psychotherapy techniques appear promising in the specific population of UM, and may require further standardization and assessment.

Working with families is a valuable aspect of UM’s experience of MUCTUM. While UMs are perceived as isolated in the host country, they are often burdened by various family problems, fears, and requests, or traumatized by a complete separation from their families or the loss of relatives. Our participants reported that care providers’ calls to their relatives to support and improve their relationship contributed greatly to their well-being.

Limitations. Our study possesses several strengths and limitations. The qualitative design allows in-depth analysis of participants’ unique experiences, which helps refine a patient-centered and flexible therapy for the culturally and psychologically heterogeneous population of UMs. Nonetheless, our participants faced challenges that impaired their ability to express themselves, resulting in fragmented answers that occasionally necessitated rephrasing as written text instead of direct quotations: Our participants were young people, were non-native speakers (some of them wanted to speak French despite the presence of an interpreter), had psychotrauma, and often had limited educational attainment. These barriers to fluid communication raise the question of how to most accurately represent the perspectives of populations who may produce disjointed discourse within current qualitative research methods. Additionally, this study does not assess MUCTUM’s efficacy. This objective could be addressed with a randomized, comparative, patient-goal-centered quantitative study, designed for a small population (i.e. using Bayesian statistics). Until then, the results of this qualitative study cannot be generalized. Another limitation is that no Asian UM consented to participate in the study, as most of them stated that they were afraid of speaking of bad memories. Future studies could determine more effective ways of recruiting Asian participants. Finally, some interviews were conducted within the hospital. Participants may not have dared to disclose negative opinions of MUCTUM in this setting.

## Conclusion

Multi-modal Co-Therapy for Unaccompanied Minors (MUCTUM) aims to provide a pluralist approach in which care providers address biological, transcultural, institutional and narrative approaches. Our qualitative study analyzes the experiences of UM participating in this program, with main findings highlighted in Table 3. We identify possible strategies to address UMs’ needs according to their own priorities, including creating a safe place, providing a patient-centered therapy, addressing concrete problems, improving relations with caseworkers, making use of *limited reparenting* in therapy, providing culturally appropriate care, and supporting family relationships. Inquiring about traumatic events may be beneficial only after these needs have been addressed.

**Table 3:** Highlights: multi-modal co-therapy for unaccompanied minors

● Commitment and reliability of care providers despite missed appointments helps build trust
● Care providers address unaccompanied minor’s needs hierarchically (e.g. housing first)
● An interpreter in native language is central for translation and cultural mediation
● Supporting the relationship with the family is experienced as helpful (e.g. through phone calls)
● Pharmacological treatment, and addressing social factors are perceived as helpful
● Therapy focuses on trauma once primary needs have been addressed. The process may take months
● Trauma narration is delayed, prudent, non-forced, and fragmented. If guided carefully, trauma narration is perceived as “freeing” by unaccompanied minors

## Supplementary Information

**Additional file 1.** COREQ (COnsolidated criteria for REporting Qualitative research) Checklist.

## Data Availability

The data that support the findings of this study are available from the corresponding author, upon reasonable request.
